# Lack of association between Fas **rs180082** polymorphism and risk of cervical cancer: an update by meta-analysis

**DOI:** 10.1186/1471-2350-14-71

**Published:** 2013-07-16

**Authors:** Xu Chen, Wuning Mo, Qiliu Peng, Xiandu Su

**Affiliations:** 1 Department of Clinical Laboratory, First Affiliated Hospital of Guangxi Medical University, 22 Shuangyong Road, Nanning 530021, Guangxi, People’s Republic of China; 2 Department of Clinical Laboratory, Nongken Nada Hospital, Nada 571700 Hainan, People’s Republic of China

**Keywords:** Fas, Cervical cancer, Polymorphism, Meta-analysis

## Abstract

**Background:**

The Fas **rs180082** polymorphism has been reported to be associated with cervical cancer susceptibility, yet the results of these previous results have been inconsistent or controversial. The objective of this study was to explore whether the Fas **rs180082** polymorphism confers susceptibility to cervical cancer.

**Methods:**

The relevant studies were identified through a search of PubMed, Excerpta Medica Database (Embase), Elsevier Science Direct and Chinese Biomedical Literature Database (CBM) until July 2012. The association between the Fas **rs180082** polymorphism and cervical cancer risk was assessed by odds ratios (ORs) together with their 95% confidence intervals (CIs).

**Results:**

A total of 7 case–control studies were eventually identified. We found no association between Fas **rs180082** polymorphism and cervical cancer susceptibility in overall population (G versus A: OR = 1.03, 95% CI = 0.99-1.07, P = 0.197; AG + GG versus AA: OR = 1.04, 95% CI = 0.98-1.09, P = 0.176; GG versus AA + AG: OR = 1.04, 95% CI = 0.84–1.31, P = 0.701). In subgroup analysis, similar results were found in Asian (G versus A: OR = 1.06, 95% CI = 0.97–1.15, P = 0.195; AG + GG versus AA: OR = 1.08, 95% CI = 0.98–1.19, P = 0.176; GG versus AA + AG: OR = 0.97, 95% CI = 0.51–1.84, P = 0.935) and African (G versus A: OR = 1.01, 95% CI = 0.97-1.15, P = 0.195; AG + GG versus AA: OR = 0.99, 95% CI = 0.91–1.07, P = 0.739; GG versus AA + AG: OR = 1.09, 95% CI = 0.94–1.25, P = 0.745).

**Conclusion:**

This meta-analysis has shown that there is a lack of association of the Fas **rs180082** polymorphisms with cervical cancer susceptibility. However, larger scale primary studies with the consideration of gene–gene and gene–environment interactions are still required to further evaluate the interaction of Fas rs180082 polymorphism with cervical cancer susceptibility.

## Background

In recent years, cervical cancer has been the second frequent women malignancy worldwide, with an estimated global incidence of 493,243 new cases and 273,505 deaths per year [1]. Therefore, it has been advanced and it is particularly necessary to explain the etiology of cervical cancer.

It is widely accepted that specific human papillomavirus (HPV) types are the central etiologic agent of cervical carcinogenesis. Other environmental and host factors also play important roles in the persistence of HPV infection and further malignant conversion of cervical epithelium [2]. A number of previous studies had suggested that the possible correlation between genetic polymorphisms of cancer susceptibility genes and the higher risk of human malignant tumors [3,4].

One of the most important mechanisms for HPV infection control of human immune system is to induce apoptosis of HPV-infected cells [5]. Apoptosis is a physiological process and it depends on signals from the cell surface death receptor Fas, which cooperates with Fas ligand (FasL) to trigger programmed cell death [6] Fas gene, which is mapped on chromosome 10q24.1, consists of nine exons and eight introns, is known as a member of tumor necrosis factor (TNF) receptors superfamily [7]. Downregulation of Fas with resultant resistance to death signals has been reported in various cancers [8-10], including cervical cancer. Since Fas has an important role in cervical cancer, any mutations in the Fas gene affecting the production of Fas may be candidate risk factors for the development of this disease. Fas has several polymorphisms such as Fas rs2234767, Fas **rs180082** and FasL rs763110. In recent years, these polymorphisms have attracted widespread attention. However, the majority of studies focused on Fas **rs180082** polymorphism.

In particular, the important polymorphism of Fas, the A/G mutation at position -670 loci, has been intensively taken into account. Furthermore, the relationship between Fas **rs180082** polymorphism and cervical cancer risk has been observed in previous studies. Although a lot of studies had shown the possible involvement of Fas **rs180082** polymorphism in the pathogenesis of cervical cancer, the results were not always consistent. Most studies showed that there was a lack of association of Fas **rs180082** polymorphism with cervical cancer risk [11-15]. However, not all the studies achieve this result [16]. Furthermore, previous meta-analysis did not include African population, which may be not comprehensive and may cause some bias to the final result [17]. Therefore, to derive a more precise estimation of the association between Fas **rs180082** polymorphism and cervical cancer risk, we performed a meta-analysis of all eligible case–control studies in this article.

## Methods

### Search strategy selection criteria

We searched electronic databases PubMed, Excerpta Medica Database (Embase), Elsevier Science Direct and Chinese Biomedical Literature Database (CBM) updated on July 2012 for all publications on the association between Fas **rs180082** polymorphism and cervical cancer susceptibility. The search strategy was based on combinations using the Mesh terms as follows: (“Fas”) in combination with (“polymorphism” or “polymorphisms” or “variant” or “SNP” or “mutation”) in combination with (“cervical cancer” or “cervical carcinoma” or “uterine cervical neoplasm”) for all publications on the association between Fas **rs180082** polymorphism and cervical cancer susceptibility. No language or country restrictions were applied. All eligible studies were retrieved, and their bibliographies were checked for other relevant publications. Review articles were also inspected to find additional eligible studies. For the use of these clinical materials for research purposes, prior consents from the patients and approval from the Ethics Committees of the First Affiliated Hospital of Guangxi Medical University were obtained.

### Inclusion and exclusion criteria

Studies included in our meta analysis had to meet the following inclusion criteria: (a) a case–control study; (b) information on the evaluation of Fas **rs180082** polymorphisms and cervical cancer risk; (c) the papers must provide sufficient data including distribution of genotype and allele frequency; (d) when multiple publications reported on the same or overlapping data, we chose the most recent or largest population. Studies were excluded if one of the following existed: (a) studies that contained overlapping data; (b) Not offering the source of cases and controls or other essential information; (c) studies in which family members had been studied because their analysis are based on linkage considerations; (d) Reviews and repeated literature were also excluded.

### Data extraction

Two investigators (Xu Chen and Wuning Mo) independently extracted the information with the standard protocol and the result was reviewed by a third investigator (Qiliu Peng).From each study, the information we extracted the name of first author, year of publication, country of origin, ethnicity of the population studied, the number of cases and controls, allele frequency, definition of cases, and genotype distribution in cases. Different ethnic descents were categorized as White, Asian, or African. For studies including subjects of different ethnic groups, data were extracted for ethnic group whenever possible.

### Statistical analysis

Data were analyzed mainly using the STATA Software (version 9.0, Stata Corp). For each study, odds ratio (OR) and its 95% confidence interval (95% CI) were calculated to assess the association strength. Meta-analysis was performed for the polymorphisms that had been investigated in at least two studies. We examined the relationship between the allele, as well as genotypes and susceptibility to cervical cancer. The heterogeneity between the studies was assessed by the χ2-test based Q-statistic. A significant Q-statistic (P < 0.10) indicated heterogeneity among the studies, and so the summary OR estimate of each study was calculated by the random -effects model (DerSimonian and Laird method) [18]. Otherwise, the fixed-effects model (the Mantel-Haenszel method) was used [19]. We also measured the effect of heterogeneity by another measure, I^2^ = 100*%* * (Q–df)/Q [20]. The I statistic measures the degree of inconsistency in the studies by calculating what percentage of the total variation across studies is due to heterogeneity rather than by chance [21]. The overall estimate of risk (OR) was calculated by a fixed effects model (Mantel–Haenszel) or a random effects model (DerSimonian–Laird) according to the presence (P < 0.10 or I2 > 50%) or absence (P > 0.10 and I2 < 50%) of heterogeneity, respectively.

Publication bias was observed with the funnel plot [22] using the standard error of log (OR). An asymmetric plot infers a possible publication bias. Funnel plot asymmetry was further assessed by the method of Egger linear regression test. The significance of the intercept was determined by the Student t test suggested by Egger (P< 0.05 was considered representative of statistically significant publication bias).

## Results

### Eligible studies

**Figure 1:**
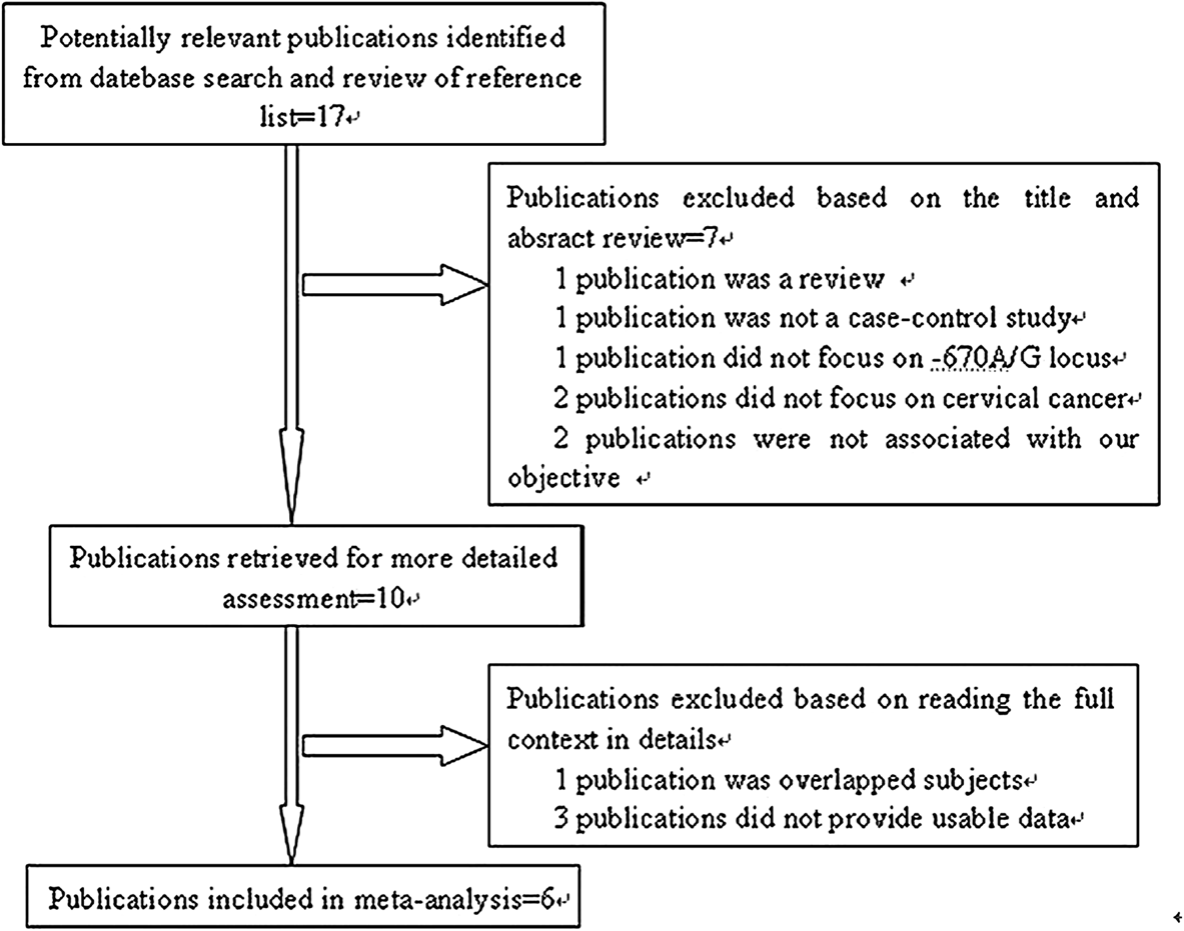
**The forest plot describing the meta-analysis with a fixed-effect recessive model (G versus A) for the association of Fas rs180082 polymorphism with cervical cancer.** Each study is depicted with size inversely proportional to its variance, accompanied by the respective 95% confidence intervals. Values of OR > 1, implied an increased risk for cervical cancer with the G allele.

Based on the search criteria, the literature search identified 17 related articles relevant to the role of the Fas **rs180082** polymorphism on cervical cancer susceptibility. Eleven of these articles were excluded: one of these articles was a review [23], one was an overlapped subject [24], one was not a case–control study [25], one did not focus on the locus that we study [26], two focused on the subjects that were not relevant to cervical cancer [27,28], two were not associated with the aim of our study [29,30], and three did not provide enough allele or genotyping data [31-33]. Manual search of references cited in the published studies did not reveal any additional articles. As a result, a total of 6 relevant studies met the inclusion criteria for the meta-analysis [11-16]. A flow diagram of the search process is shown in Figure 1. Therefore, a total of 7 separate case–control studies, consisting of 1,856 cervical cancer patients and 2,097 controls were included in our meta-analysis ([11] literature has two case–control studies). The selected study characteristics were listed in Table 1. There were 4 case–control studies of subjects of Asian descent [12-14,16], 2 case–control studies of subjects of African descent [11] and one case–control study of subject of White descent [15]. The genotype frequencies for control group in two studies were not consistent with Hardy-Weinberg equilibrium (HWE) [11,13].

**Table 1:** Main characteristics of studies included in the meta-analysis

**First author**	**Year**	**Country**	**Ethnicity**	**Genotyping method**	**Source of control**	**Cases/controls**	**HWE-control**
Li H	2009	China	Asian	PCR-PFLP	HB	314/615	0.640
Kordi T	2008	India	Asian	PCR-PFLP	HB	200/200	0.000
Sokbom K	2007	South Korea	Asian	PCR-PFLP	HB	154/160	0.264
Ueda M	2006	Japan	Asian	PCR-PFLP	HB	83/95	0.172
Zoodsma M	2005	Netherlands	White	Taq Man	PB	670/607	0.274
Kooshik C	2009	South Africa	African	Taq Man	HB	106/101	0.047
Kooshik C	2009	South Africa	African	Taq Man	HB	341/323	0.985

*PCR-RFLP* PCR-restriction fragment length polymorphism, *HWE* Hardy-Weinberg equilibrium, *HB* hospital based, *PB* population based.

### Quantitative synthesis of data

**Figure 2:**
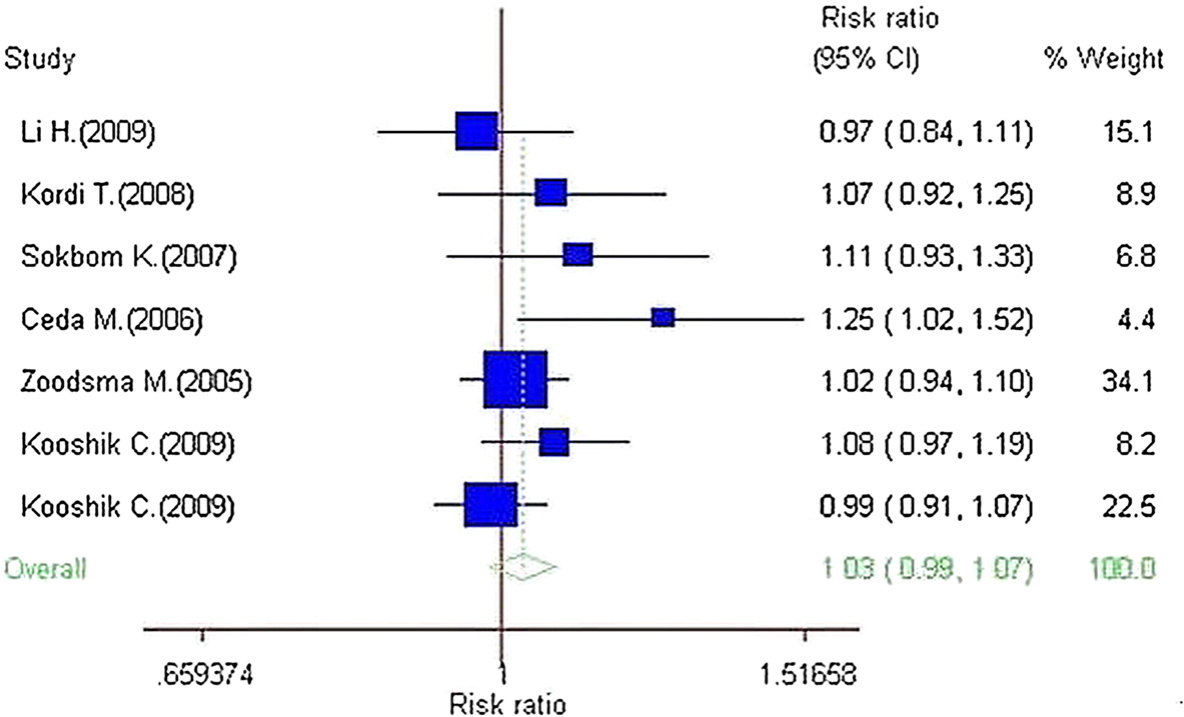
**Forest plot of cervical cancer risk associated with Fas rs180082 (G versus A).** The squares and horizontal lines correspond to the study-specific OR and 95% CI. The area of the squares reflects the study-specific weight (inverse of the variance). The diamond represents the summary OR and 95% CI.

Summary results of this meta-analysis for the association between Fas **rs180082** polymorphism and cervical cancer risk are shown in Table 2. The meta-analysis showed no association between cervical cancer and the Fas -670G allele in the overall population (OR=1.03, 95% CI= 0.99-1.07, P=0.197; Figure 2). The overall OR for the GG+GA genotype of the Fas -670 was 1.04 in the overall population (95% CI=0.98-1.09, P=0.176). The overall OR for the GG genotype of the Fas -670 was 1.04 in the overall population (95% CI=0.84-1.31, P=0.701). Stratification by ethnicity revealed still no association of the G allele, and the GG and the GG + GA genotypes with cervical cancer in Asian and African (Table 2).

**Table 2:** Results of meta-analysis for the Fas rs180082 polymorphism and cervical cancer risk

**Comparison**	**Population**	**N**	**Sample size**		**Test of association**			**Mode**	**Test of heterogeneity**		
			**Case**	**Control**	**OR**	**95% CI**	**P**		**χ2**	**P**	**I**^**2**^
G vs. A	Overall	7	3712	4194	1.03	0.99-1.07	0.197	F	7.38	0.288	18.6
	Asian	4	1502	2140	1.06	0.97-1.15	0.195	F	4.76	0.190	37.0
	Afrian	2	870	840	1.01	0.95-1.08	0.776	F	1.88	0.170	46.9
GG+GA vs. AA	Overall	7	1856	2097	1.04	0.98-1.09	0.176	R	17.75	0.007	66.2
	Asian	4	751	1070	1.08	0.98-1.19	0.125	R	6.90	0.075	56.5
	Afrian	2	435	420	0.99	0.91-1.07	0.739	R	5.65	0.017	82.3
GG vs. GA+AA	Overall	7	1856	2097	1.04	0.84-1.31	0.701	F	21.56	0.001	72.2
	Asian	4	751	1070	0.97	0.51-1.84	0.935	R	18.34	0.000	83.6
	Afrian	2	435	420	1.09	0.94-1.25	0.245	F	0.82	0.364	0.0

*OR* odds ratio, *CI* confidence interval, *F* fixed effects model, *R* random effects model.

### Sensitivity analysis

Although 2 studies [11,13] did not follow the HWE, the summary ORs were not effectively altered including or without including the studies. Moreover, no other single study influenced the overall results qualitatively as indicated by sensitivity analysis.

### Test of heterogeneity

There was significant heterogeneity for heterozygote comparison of dominant model comparison (GA+AA vs. GG: P = 0.007) and recessive model comparison (GG vs. GA+AA: P= 0.001). Then, we assessed the source of heterogeneity for heterozygote comparison (GA+AA vs. GG) by ethnicity. As a result, ethnicity (χ2 = 14.41; df = 2; P = 0.001) was found to contribute to substantial heterogeneity.

### Publication bias

**Figure 3:**
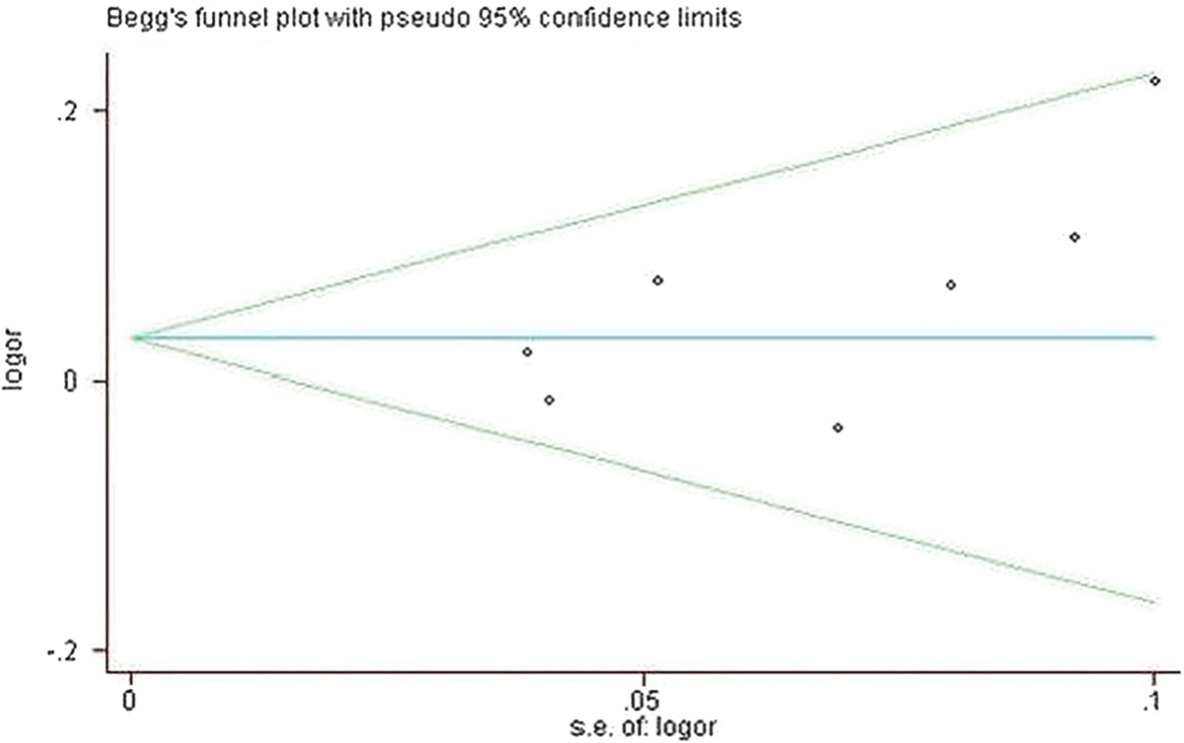
**Begg funnel plot for publication bias test (G versus A).** Each point represents a separate study for the indicated association. Log [OR], natural logarithm of OR. Horizontal line means effect size.

Begg funnel plot was created to assess the publication bias of literatures. As shown in Figure 3, the shapes of the funnel plots did not reveal any evidence of obvious asymmetry. Then, the Egger test was used to provide statistical evidence of funnel plot symmetry. The results still also suggested the absence of publication bias (t=1.93; P=0.112 for G vs. A).

## Discussion

The alterations of apoptosis-related genes are probably contributing to malignant tumors, including cervical cancer [34]. Fas is an important apoptosis-related gene and known as a member of tumor necrosis factor (TNF) receptors superfamily [7]. Downregulation of Fas with resultant resistance to death signals has been reported in many cancers [23]. Furthermore, the transcript expression of Fas is modulated by genetic elements which located in the 5*′* upstream promoter region of the gene. SNP at -670 in the enhancer region (A/G) locates at a binding element of gamma interferon activation signal (GAS). Homozygous for G allele (GG) could lead to a complete deletion of the binding sequence of transcription element GAS, which is responsible for the signal emanated through STAT1, and in a significant alteration in the gene expression [35].

Although previous meta-analysis maybe involves some parts of the relationship between Fas **rs180082** polymorphism and cervical cancer risk, its eligible studies are not quite comprehensive [17]. First, lack of the population of African, which leads to the decrease of studies and may cause a deviation to final result; second, some of the its eligible studies regard squamous cell carcinoma (SCC) or squamous intraepithelial lesion (SIL) as the cervical cancer [29,33]. However, SCC is just one pathological type of cervical cancer and SIL is just a stage in the pathological process of cervical cancer, which may also bring some bias. Therefore, we conduct an update by meta-analysis to comprehensively evaluate the association between **rs180082** polymorphism and cervical cancer risk. This update meta-analysis includes seven latest case–control studies, five of which are published in recent 5 years. Despite the number of eligible studies in this meta-analysis is still infinite, all the studies included in this meta-analysis, which are based on stricter search criteria, are more reliable and lead to a more accurate result.

As for cervical cancer risk, the previous results of the studies involving Fas **rs180082** polymorphism were contradictory. These inconsistent results were possibly due to small effect of the polymorphism on cervical cancer risk or the relatively low statistical power of the published studies. Consequently, the meta-analysis was needed to provide a quantitative approach for combining the results of various studies with the same topic and for estimating and explaining their diversity.

So far, the number of the studies that focus on the relationship between Fas **rs180082** polymorphism and cancer risk is limited. Some studies before had shown that there was an association between Fas **rs180082** and some cancers, including nasopharyngeal carcinoma [36], lung cancer [37,38], gastric cancer [39] and leukemia [40]. Otherwise, some studies draw a different conclusion [41].

The studies included in our meta-analysis may differ in some aspects. Koushik, et al. conducted the first African population study with 447 women with invasive cervical cancer and 424 healthy women controls [11]. Li, et al. conducted the study with 314 women of cervical cancer and 615 healthy women controls in South China [12]. Kordi, et al. conducted the first study in North India [13]. Zoodsma, et al. and Kang, et al. conducted the same study in Netherland and South Korea [14,15]. All of them did not find any significant association between Fas **rs180082** polymorphism and cervical cancer susceptibility. However, Ueda, et al. found that polymorphism of Fas gene promoter -670 may be associated with the risk of cervical cancer in a Japanese population. It should be pointed out that the study which focused on Japanese population is based on small sample size (<200 subjects), so that we should be cautious to the result of this study. Consequently, our data failed to find a relationship between Fas **rs180082** polymorphism and cervical cancer risk. The present meta-analysis, which included 1856 cases of cervical cancer and 2097 controls, suggested that there was no association between Fas **rs180082** polymorphism and cervical cancer susceptibility.

Considering the results may be due to different ethnicity, we further conducted subgroup analysis according to ethnicity. When stratifying for ethnicity, significant association was detected in neither Asian populations nor African populations, suggesting that the genetic background or environment they live in may not influence the Fas **rs180082** polymorphism on cervical cancer susceptibility. However, the conclusion should be cautious. Because the sample size of studies included in our meta-analysis is relatively small, especially in the African population. Our results may be underpowered so that further studies need to be conducted to increase the statistical power. The genetic distance between different ethnicities considered together could be substantial. It is not excluded that true effects are present in one specific sub-population, but undetected due to lack of statistical power or diluted/cancelled out by population stratification issues when different study populations are grouped together for analysis.

Some limitations of this meta-analysis we need to pay attention to. First, our results are based on unadjusted estimates and a more precise analysis stratified by age, different lifestyle related habits and different grades of cervical cancer could be performed if individual data were available. Second, lack of the original data of the reviewed studies limited our precise estimation of the relation, which might cause some bias. Third, the lack of original study limited our further evaluation of potential interactions because the interactions between gene-environment interactions may modulate cancer risk.

Despite these limitations or disadvantages, our meta-analysis still had some advantages. First, a systematic review of the association of Fas **rs180082** polymorphism with cervical cancer risk is statistically more powerful than any single study. Second, the studies retrieved were the latest, half of which were published in the recent 3 years. Third, the quality of case–control studies included in our Meta analysis was satisfactory and met our inclusion criteria.

## Conclusions

In summary, the present meta-analysis provides information that there is a lack of association of the Fas **rs180082** polymorphisms with cervical cancer. However, larger scale primary studies with the consideration of gene–gene and gene–environment interactions are still required to further evaluate the interaction of Fas **rs180082** polymorphism with cervical cancer susceptibility.

## Competing interests

In the past five years we did not receive any reimbursements, fees, funding, or salary from an organization that may in any way gain or lose financially from the publication of this manuscript. We do not hold any stocks or shares in an organization that may in any way gain or lose financially from the publication of this manuscript. We do not hold and we are not currently applying for any patents relating to the content of the manuscript. We do not receive reimbursements, fees, funding, or salary from an organization that holds or has applied for patents relating to the content of the manuscript. There are no any non-financial competing interests (political, personal, religious, ideological, academic, intellectual, commercial or any other) to declare in relation to this manuscript.

## Authors’ contributions

XC collected the literature data, developed the statistical model, carried out the software implementation and drafted the manuscript. QP collected the literature data, read the full text articles, and checked the model and results. XS collected the literature data and read the full text articles. WM helped with the discussion both in the theoretical development and English copyediting. All authors read and approved the final manuscript.

## Pre-publication history

The pre-publication history for this paper can be accessed here:

http://www.biomedcentral.com/1471-2350/14/71/prepub
